# Effects of Capsaicin and Capsiate on Endurance Performance: A Meta-Analysis

**DOI:** 10.3390/nu14214531

**Published:** 2022-10-28

**Authors:** Jozo Grgic, Aamir Raoof Memon, Sitong Chen, Rodrigo Ramirez-Campillo, Gabriel Barreto, Markus Estifanos Haugen, Brad J. Schoenfeld

**Affiliations:** 1 Institute for Health and Sport, Victoria University, Melbourne, VIC 3011, Australia; 2 Exercise and Rehabilitation Sciences Institute, School of Physical Therapy, Faculty of Rehabilitation Sciences, Universidad Andres Bello, Santiago 7591538, Chile; 3 Applied Physiology and Nutrition Research Group, School of Physical Education and Sport, Faculty of Medicine FMUSP, University of São Paulo, São Paulo 05405-000, SP, Brazil; 4 Department of Sports Sciences and Physical Education, Nord University, 7600 Levanger, Norway; 5 Department of Health Sciences, CUNY Lehman College, New York, NY 10468, USA

**Keywords:** dietary supplement, resistance training, performance-enhancing substances

## Abstract

Several studies have explored the effects of capsaicin and capsiate on endurance performance, with conflicting findings. This systematic review aimed to perform a meta-analysis examining the effects of capsaicin and capsiate vs. placebo on endurance performance in humans. Seven databases were searched to find eligible studies. The effects of capsaicin and capsiate on aerobic endurance (e.g., time-trials or time-to-exhaustion tests), muscular endurance (e.g., repetitions performed to muscular failure), and rating of perceived exertion (RPE) were examined in a random-effects meta-analysis. Fourteen studies (*n* = 183) were included in the review. Most studies provided capsaicin or capsiate in the dose of 12 mg, 45 min before exercise. In the meta-analysis for aerobic endurance, there was no significant difference between the placebo and capsaicin/capsiate conditions (Cohen’s *d*: 0.04; 95% confidence interval: −0.16, 0.25; *p* = 0.69). In subgroup meta-analyses, there were no significant differences between the placebo and capsaicin/capsiate conditions when analyzing only studies that used time-trials (*p* = 0.20) or time-to-exhaustion tests (*p* = 0.80). In the meta-analysis for muscular endurance, a significant ergogenic effect of capsaicin/capsiate was found (Cohen’s *d*: 0.27; 95% confidence interval: 0.10, 0.43; *p* = 0.002). When analyzing set-specific effects, an ergogenic effect of capsaicin/capsiate was found in set 1, set 2, and set 3 (Cohen’s *d*: 0.21–29). Capsaicin/capsiate ingestion reduced RPE following muscular endurance (*p* = 0.03) but not aerobic endurance tests (*p* = 0.58). In summary, capsaicin/capsiate supplementation acutely enhances muscular endurance, while the effects on aerobic endurance are less clear.

## 1. Introduction

Capsaicin is a natural substance primarily found in chili peppers and other spicy foods [1]. Capsaicin is one of the pungent compounds in a group of capsaicinoids; other compounds include dihydrocapsaicin and nordihydrocapsaicin [1]. About 70% of the burn of hot red peppers is attributed to capsaicin [1,2]. In contrast, CH-19 Sweet (*Capsicum annuum* L.) is a non-pungent red pepper variety that contains capsinoids [2,3]. These are non-pungent capsaicin analogs and, among others, include capsiate. Capsaicin and capsiate have a very similar chemical structure [1,2]. They only vary at the center linkage, where capsaicin and capsiate contain amide and ester bonds, respectively [1,2].

Both capsaicin and capsiate selectively bind to transient receptor potential vanilloid subtype 1 (TRPV1) [1]. The differences in pungency between capsaicin and capsiate are related to the site of TRPV1 activation. While capsaicin activates TRPV1 in the tongue, capsiate is hydrolyzed as it crosses the oral mucosa [1]. Due to their effects on TRPV1 activation, capsaicin and capsiate have also received attention among researchers in sport and exercise science [4]. Activation of TRPV1 leads to the sensation of heat and an increase in energy expenditure and fat oxidation [1]. Furthermore, TRPV1 is also highly expressed in skeletal muscle (located on the sarcoplasmic reticulum) and its activation may also influence muscle contractility [5,6]. TRPV1 activation following capsaicin ingestion may increase calcium ion release from the sarcoplasmic reticulum, subsequently influencing the interaction between the actin-myosin filaments resulting in an increase in force output [5,6]. Consumption of capsaicin has also been reported to reduce pain perception and rating of perceived exertion (RPE), factors that may contribute to improvement in exercise performance [4,7]. Activation of TRPV1 may also stimulate nitric oxide synthesis, a potent vasodilator [8]. Vasodilatation may enhance exercise performance by increasing blood flow, nutrient and oxygen delivery to the working muscle, and the clearance of metabolic by-products [9]. 

Due to these physiological effects, studies have explored the effects of capsaicin and capsiate on exercise performance. Much of the early research on the topic was performed using animal models [10,11]. For example, one study explored the effects of 10 mg/kg or 15 mg/kg of capsaicin on swimming endurance in mice [11]. The consumption of 10 mg/kg of capsaicin enhanced endurance performance and these effects were largely attributed to the increase in fatty acid utilization [11]. Deriving from these findings in animal models, recent studies have explored the effects of capsaicin and capsiate on exercise performance in humans [12,13,14,15,16,17,18,19,20,21,22,23,24,25]. One of the initial studies investigated the effects of capsaicin on muscular endurance in resistance exercise, using a protocol involving four sets performed to muscular failure with 70% of one-repetition maximum (1RM) in the squat [14]. The consumption of 12 mg of capsaicin, 45 min before exercise, increased total volume load performed in the four sets [14]. Other studies used a very similar protocol of supplementation and exercise tests and did not observe an ergogenic effect of capsaicin [23]. Capsaicin or capsiate ingestion has also been reported to enhance aerobic endurance in different cycling or running tasks, even though this finding is not consistent in the literature [15,19].

A recent review evaluated the effects of capsaicin and capsiate on exercise performance and concluded that they might provide an ergogenic effect [4]. However, this review was performed narratively, and it lacked a meta-analysis that would allow the pooling of outputs from different studies on a given topic. Such an analysis would be valuable given that some studies might have been underpowered to detect a significant effect. Additionally, this area of research is growing, as evidenced by the publication of seven studies on the effects of capsaicin and capsiate on endurance performance in 2021 and 2022 [17,18,19,20,23,24,25]. Due to the growing evidence and the conflicting findings, we aimed to perform a systematic review and meta-analysis exploring the effects of capsaicin and capsiate on endurance performance in humans. 

## 2. Materials and Methods

### 2.1. Search Strategy

We followed the PRISMA guidelines for this review [26]. The search for studies was conducted in two phases. The first phase of the search process involved examining the available literature in different bibliographic databases, including Academic Search Elite, Networked Digital Library of Theses and Dissertations, Open Access Theses and Dissertations, PubMed/MEDLINE, Scopus, SPORTDiscus, and Web of Science. The following search syntax was utilized: (capsaicin OR capsiate) AND (“muscular endurance” OR “resistance training” OR “resistance exercise” OR “time-to-exhaustion” OR “aerobic endurance” OR “time-trial” OR “exercise performance”). After examining the search results in the databases, the second phase of the search process was performed. This phase was comprised of reviewing the reference lists of the included studies (backward citation tracking) and studies that cited the included studies (forward citation tracking) in Google Scholar. The search process for this review was performed on 15 July 2022. The search was performed independently by two authors of the review (JG and SC) to reduce the likelihood of selection bias. 

### 2.2. Inclusion Criteria

Studies were included in the review if they satisfied the following PICO criteria:

- Population (P): healthy adult human participants
- Interventions (I): acute, pre-exercise, capsaicin and/or capsiate supplementation
- Comparison (C): placebo
- Outcome (O): aerobic endurance (e.g., cycling time-trial) or muscular endurance performance (e.g., repetitions performed to muscular failure)

We also required the studies to be published in English as a journal article, thesis, or dissertation.

### 2.3. Data Extraction

From each of the included studies, the following data were extracted:

- Lead author name and year of study publication
- Study design
- Participants’ characteristics (e.g., sex, training status)
- Supplementation protocols
- Description of the performance test
- Mean ± standard deviation performance and RPE data recorded in the placebo and experimental trials

### 2.4. Risk of Bias Assessment

Evaluation of the risk of bias in the included studies was performed using RoB 2 tool, with additional considerations for studies using a crossover design [27]. The RoB 2 tool evaluates the risk of bias in six domains, including: domain 1—bias arising from the randomization process; domain S—bias arising from period and carryover effects; domain 2—bias due to deviations from intended intervention; domain 3—bias due to missing outcome data; domain 4—bias in the measurement of the outcome; domain 5—bias in the selection of the reported result. Evaluation of each of these six domains and the overall risk of bias is categorized as “low risk”, “some concerns” or “high risk”, in accordance with the RoB 2 tool recommendations [27]. Risk of bias evaluation was performed independently by two authors of the review (JG and ARM).

### 2.5. Statistical Analysis

The mean ± standard deviation performance and RPE data recorded during the placebo and capsaicin/capsiate conditions were converted to standardized mean differences (Cohen’s *d* effect size) and pooled in a random-effects meta-analysis. Effect sizes were calculated as the difference in means divided by the pooled standard deviation. The calculation of effect sizes and their respective 95% confidence intervals (CI) was performed using the performance data, sample size, and the correlation between trials. None of the included studies presented the correlation between trials. Therefore, for the analysis of aerobic endurance, correlation values were calculated using the individual data presented in one study [12]. In this study, *r* amounted to 0.90 and therefore this value was used for all studies in the aerobic endurance analysis. For the analysis of muscular endurance, correlation was calculated using the individual data from two studies [20,23]. In these two studies, correlation values were 0.65 and 0.93. The average between these two values (*r* = 0.79) was used for all studies included in the analysis of muscular endurance.

In the main meta-analyses, placebo was compared with capsaicin/capsiate. Both capsaicin and capsiate were considered in the same analysis given that: (a) capsiate is a capsaicin analog and these compounds only differ in pungency; (b) both have a very similar chemical structure; and (c) their mechanism of action is related to the activation of TRPV1 [1,2,3,4]. However, sensitivity analyses were also performed by excluding the data from studies that provided capsiate supplementation. This analysis allowed the isolation of capsaicin’s effects, which is a compound more often used in studies on exercise performance. For aerobic endurance, a subgroup meta-analysis was performed to explore the effects of capsaicin/capsiate in time-trials vs. time-to-exhaustion tests. For muscular endurance, a subgroup meta-analysis was performed to explore set-specific effects of capsaicin/capsiate; namely, separate effects were calculated for performance in sets 1, 2, and 3. 

Effect sizes were interpreted using the established thresholds: trivial (<0.20), small (0.20–0.49), medium (0.50–0.79), and large (≥0.80) [28]. *I*^2^ statistic was used to examine heterogeneity and interpreted as low (<50%), moderate (50–75%), and high heterogeneity (>75%). The statistical significance threshold was set at *p* < 0.05. All analyses were performed using the Comprehensive Meta-Analysis software, version 2 (Biostat Inc., Englewood, NJ, USA).

## 3. Results

### 3.1. Search Results

**Figure 1:**
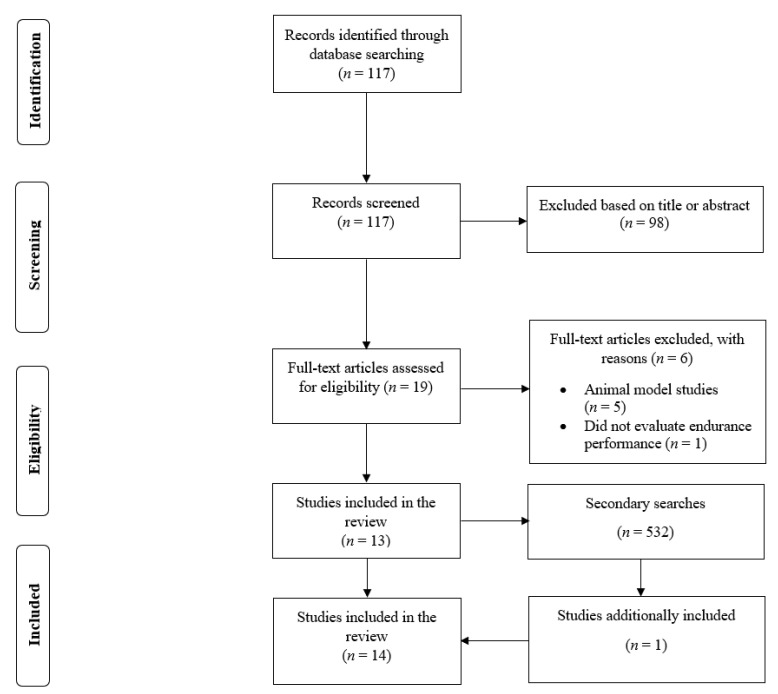
Flow diagram of the search process.

In the first phase of the search process, there were 117 search results. Of these, 98 were excluded based on title or abstract. Therefore, 19 full-text papers were assessed for eligibility. Six studies were excluded because they were performed using animal models or did not evaluate endurance performance. Thus, 13 studies were found to satisfy the inclusion criteria in the first phase [12,13,14,15,16,17,18,19,20,21,22,23,25]. In the second phase, an additional 532 search results were found, and one study satisfied the inclusion criteria [24]. After completing both phases of the search process, 14 studies were included for analysis in this review (Figure 1) [12,13,14,15,16,17,18,19,20,21,22,23,24,25]. 

### 3.2. Summary of the Included Studies

Seven studies with a total of 94 participants (5 females and 89 males) evaluated aerobic endurance [12,15,16,19,21,22,25]. Five studies provided capsaicin supplementation in doses of 1.2 mg, 7.8 mg, or 12 mg, 45-50 min before exercise. Two studies provided 12 mg of capsiate, 45 min before exercise. Four studies used running or cycling to exhaustion tests, while three used time-trials (Table 1). All seven studies evaluated RPE.

Seven studies with a total of 89 participants (5 females and 84 males) evaluated muscular endurance [13,14,17,18,20,23,24]. Capsaicin supplementation was provided in five studies, which used doses of 1.2 mg, 12 mg, or 24 mg, provided 45 min before exercise. Two studies provided 6 or 12 mg of capsiate, 45 min before exercise. Five studies evaluated muscular endurance using several sets of resistance exercise (squat, leg extension, or bench press) performed to muscular failure with 70% of 1RM. One study used 120 maximal isokinetic knee extensions, while one used multiple sets of 10-s isometric contractions (Table 1). Four studies evaluated RPE. 

Regardless of the outcome, studies most commonly provided supplementation in capsule form. Two studies, however, provided supplementation in chewable form [13,21]. In most studies, the content of the placebo was starch, while some studies also used psyllium husk or maltodextrin.

### 3.3. Risk of Bias

For domains 1, S, 2, 4 and 5, the classification for the included studies was either “low risk” or “some concerns”. However, for domain 3, studies scored “low risk”, “some concerns” or “high risk” of bias. The overall evaluation of risk of bias on the RoB 2 tool for all included studies was “some concerns” or “high risk” of bias (Table 2).

### 3.4. Meta-Analysis for Aerobic Endurance

**Figure 2:**
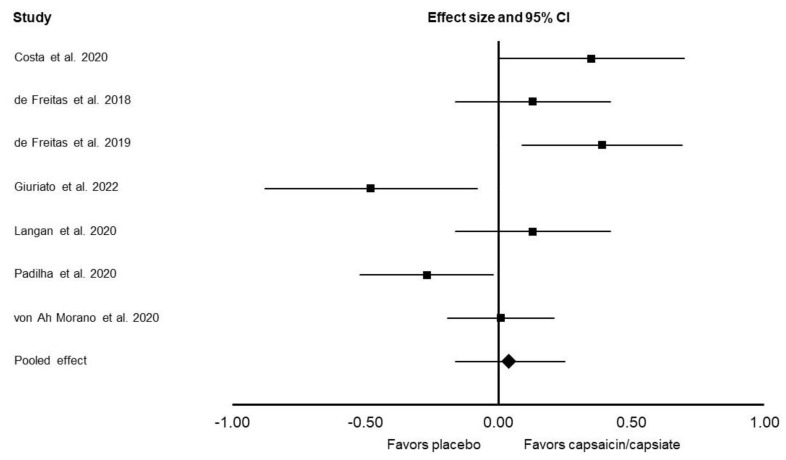
Forest plot presenting the results of the random-effects meta-analysis, which explored the effects of capsaicin/capsiate vs. placebo on aerobic endurance. Data are presented as effect sizes (Cohen’s *d*) and 95% confidence interval (CI). The diamond at the bottom presents the overall effect. The plotted squares denote effect sizes, and the whiskers denote their 95% CIs. The analysis is based on seven included studies [12,15,16,19,21,22,25].

For aerobic endurance, there was no significant difference between the placebo and capsaicin/capsiate conditions (Cohen’s *d*: 0.04; 95% CI: −0.16, 0.25; *I*^2^ = 72%; *p* = 0.69; Figure 2). These results were minimally altered after the exclusion of the two studies that provided capsiate supplementation (Cohen’s *d*: −0.01; 95% CI: −0.30, 0.28; *I*^2^ = 78%; *p* = 0.94). In subgroup meta-analyses, there was no significant difference between the placebo and capsaicin/capsiate conditions when analyzing only studies that used time-trials (Cohen’s *d*: 0.12; 95% CI: −0.06, 0.30; *I*^2^ = 29%; *p* = 0.20), or time-to-exhaustion tests (Cohen’s *d*: −0.05; 95% CI: −0.41, 0.32; *I*^2^ = 83%; *p* = 0.80). There was no significant difference between placebo and capsaicin/capsiate conditions for RPE (Cohen’s *d*: 0.07; 95% CI: −0.18, 0.32; *I*^2^ = 81%; *p* = 0.58).

### 3.5. Meta-Analysis for Muscular Endurance

**Figure 3:**
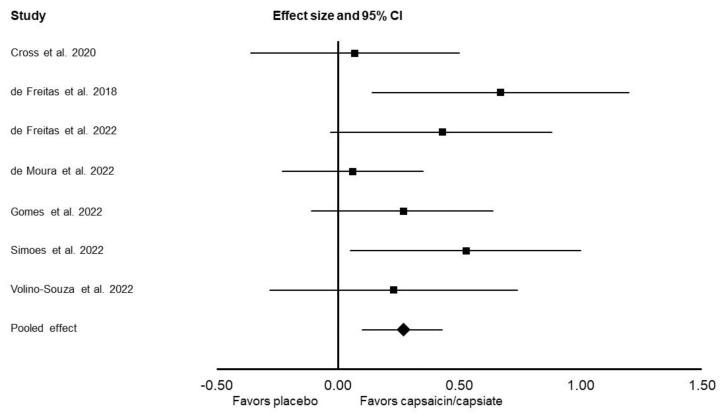
Forest plot presenting the results of the random-effects meta-analysis, which explored the effects of capsaicin/capsiate vs. placebo on muscular endurance. Data are presented as effect sizes (Cohen’s *d*) and 95% confidence interval (CI). The diamond at the bottom presents the overall effect. The plotted squares denote effect sizes, and the whiskers denote their 95% CIs. The analysis is based on seven included studies [13,14,17,18,20,23,24].

For muscular endurance, a significant ergogenic effect of capsaicin/capsiate was found (Cohen’s *d*: 0.27; 95% CI: 0.10, 0.43; *I*^2^ = 9%; *p* = 0.002; Figure 3). An ergogenic effect was also found when excluding the data from the two studies that provided capsiate supplementation (Cohen’s *d*: 0.36; 95% CI: 0.15, 0.58; *I*^2^ = 8%; *p* = 0.002). When analyzing set-specific effects, an ergogenic effect of capsaicin/capsiate was found in set 1 (Cohen’s *d*: 0.21; 95% CI: 0.03, 0.38; *I*^2^ = 0%; *p* = 0.02), set 2 (Cohen’s *d*: 0.20; 95% CI: 0.01, 0.39; *I*^2^ = 11%; *p* = 0.04), and set 3 (Cohen’s *d*: 0.29; 95% CI: 0.06, 0.52; *I*^2^ = 39%; *p* = 0.01). Capsaicin/capsiate significantly reduced RPE (Cohen’s *d*: −0.48; 95% CI: −0.91, −0.05; *I*^2^ = 75%; *p* = 0.03).

## 4. Discussion

This is the first meta-analysis that explored the effects of capsaicin and capsiate on endurance performance. Fourteen studies were included in the review, most of which provided capsaicin or capsiate in a dose of 12 mg, 45 min before exercise. The main finding of this review is that capsaicin/capsiate ingestion is ergogenic for muscular endurance. These ergogenic effects were present even when isolated capsaicin consumption was considered in the analysis. Additionally, improvements in muscular endurance do not seem to be set-specific, given that an ergogenic effect was found in all analyzed sets (i.e., sets 1, 2, and 3). The improvements in muscular endurance appear to be mediated by reductions in RPE. While an ergogenic effect was found for muscular endurance, there was no significant difference between the placebo and capsaicin/capsiate conditions for aerobic endurance.

In the aerobic endurance analysis, there were no significant differences between the placebo and capsaicin/capsiate conditions. The pooled effect size and its 95% CI (Cohen’s *d*: 0.04; 95% CI: −0.16, 0.25) were in the range of a trivial to small effect. This suggests that even if there is an actual effect of capsaicin/capsiate on aerobic endurance performance, it would only be trivial or small [28]. While there were no significant differences between the conditions, moderate to high heterogeneity (*I*^2^ = 72% to 78%) was observed. To explore potential reasons for this heterogeneity, subgroup analyses were performed, which showed that *I*^2^ was much lower in the analysis for time-trials vs. time-to-exhaustion tests (29% vs. 80%). Previous studies explored the test-retest reliability of time-trials and time-to-exhaustion tests and demonstrated that time-trials have a much lower coefficient of variation, indicating greater reliability [29]. It might be that the use of time-to-exhaustion tests in some studies was associated with poor test-retest reliability and thus increased type II errors [29]. Indeed, out of the four studies that used time-to-exhaustion tests, only one found an ergogenic effect [16,19,21,22]. In contrast, two of the three studies using time-trials reported ergogenicity associated with capsaicin or capsiate consumption [12,15,25]. Thus, future studies are needed to explore the influence of capsaicin and capsiate on endurance performance using time-trials and time-to-exhaustion tests to examine if their effects might be test-specific.

Besides methodological aspects, the physiological influence of capsaicin and capsiate ingestion should be considered. Specifically, a reduction in RPE was suggested to contribute to the ergogenic effect of capsaicin and capsiate on endurance performance [4]. However, this analysis did not find any alterations in RPE values, which might also explain the lack of an ergogenic effect. Additionally, capsaicin and capsiate are suggested to increase fat oxidation during exercise, which could lead to glycogen sparing [1]. One of the included studies provided a detailed assessment of various physiological parameters (e.g., oxygen consumption during and 20-min post-exercise, energy systems contribution, heart rate, RPE) and reported that none of them were affected by capsaicin ingestion [22]. The lack of physiological alterations following supplementation might also explain why there were no performance improvements in this analysis. While the present analysis did not find an ergogenic effect, it might be that a different protocol of capsaicin and capsiate ingestion would be ergogenic. For example, capsaicin ingestion has been reported to increase fat oxidation when using a daily dose of 135 mg, which is much larger than the most common dose of 12 mg used in the included studies [30]. Future research is needed to explore the influence of capsaicin and capsiate dose and supplementation protocol (i.e., acute vs. chronic) on aerobic endurance.

An ergogenic effect of capsaicin/capsiate supplementation was found in the analysis for muscular endurance. When excluding the data from studies providing capsiate supplementation, the ergogenic effect was still present and even slightly increased (Cohen’s *d*: 0.27 vs. 0.36). There are several possible physiological explanations for the ergogenic effect of capsaicin/capsiate on muscular endurance. As mentioned previously, the activation of TRPV1 may lead to an increase in calcium ion release in the sarcoplasmic reticulum, thus influencing the interaction between the actin-myosin filaments and enhancing force output [5,6]. Capsaicin and capsiate may alter motor neuron excitability and motor unit recruitment [31]. These supplements may also reduce RPE during resistance exercise—another factor contributing to improved performance. Indeed, a meta-analysis was also performed for RPE, which found that capsaicin/capsiate supplementation significantly reduced RPE (Cohen’s *d*: −0.48). These results suggest that a reduction in RPE might be a significant contributor to the mechanisms underpinning the ergogenic effect [4]. Capsaicin binds to nociceptors, the sensory receptors that send signals influencing pain perception [7]. Subsequently, afferents III and IV respond to TRPV1 agonists [32,33], such as capsaicin and capsiate, thus reducing levels of exercise-related discomfort [34]. This effect of capsaicin on nociceptors conceivably explains why its ingestion is also associated with a reduction in RPE. While these proposed factors may explain the observed ergogenic effect, future work on the mechanisms underpinning the performance improvement following capsaicin and capsiate supplementation is certainly needed.

Most of the included studies evaluated the effect of capsaicin and capsiate on muscular endurance using a protocol involving multiple sets. The outcome variable in these studies was volume load (sets × repetitions × load) or the number of repetitions performed throughout all sets. While this approach is justified in evaluating the amount of work performed, it does not provide insights into the possible set-specific effects of capsaicin and capsiate. In the present review, a meta-analysis was also performed to explore the effects of capsaicin/capsiate across different sets, observing similar improvements in sets 1, 2, and 3 (Cohen’s *d*: 0.21 to 0.29). Even though the number of performed repetitions decreased with each subsequent set (12–17 repetitions in set 1, 8–11 repetitions in set 2, and 6–9 repetitions in set 3), these results suggest that the ergogenicity of capsaicin and capsiate might not increase alongside fatigue accrued during exercise. Based on the results, capsaicin/capsiate supplementation may be considered in single and multiple-set resistance training protocols [35].

The magnitude of improvements in muscular endurance observed following capsaicin/capsiate ingestion is very similar to that observed with the ingestion of other supplements. For example, pooled Cohen’s *d* for the effect of caffeine on muscular endurance is reported to be between 0.25 and 0.38 [36,37,38]. Sodium bicarbonate also enhances muscular endurance [39,40]. Specifically, a recent meta-analysis reported an effect size of 0.37 for sodium bicarbonate supplementation, which is similar to the pooled effects reported herein [39]. Besides caffeine and sodium bicarbonate, an ergogenic effect on muscular endurance was also observed following dietary nitrate ingestion (Cohen’s *d*: 0.31) [41]. Given these comparable ergogenic effects, future studies may consider exploring the combined effects of capsaicin and capsiate with other ergogenic aids such as caffeine, sodium bicarbonate, and dietary nitrate. This would be relevant given that athletes commonly use several supplements to maximize performance gains, and different ingredients are combined in pre-workout supplements [42].

While this review has strengths, such as performing the search for studies in seven databases indexing published and unpublished documents (i.e., peer-reviewed and grey literature) and the comprehensive forward and backward citation tracking, several limitations also need to be acknowledged. Three studies [13,19,21] used a single-blind design, which inherently offers evidence of lower methodological quality. While all studies blinded the participants, none evaluated the effectiveness of this blinding. This procedure should be incorporated into future studies given that correct supplement identification may influence the outcome of an exercise test and lead to bias in the results [43]. This aspect may be especially relevant when providing supplementation in gum form, for which participants have noted that the taste is more pungent than the placebo comparison [13]. The findings presented herein are mostly specific to male participants, given that ∼95% of participants in the analysis for both endurance outcomes were men. Additionally, participants in all analyzed studies were healthy young adults. Thus, these results may not necessarily be generalized to older adults and those with various health conditions. Future studies may consider exploring the effects of capsaicin/capsiate in these populations. The influence of capsaicin and capsiate dose could not be explored using a meta-regression, given that most studies used a dose of 12 mg provided 45 min before exercise. Future studies are needed to explore the influence of capsaicin and capsiate dose on endurance performance, given that differences in doses between the studies (e.g., 1.2 mg vs. 12 mg) may explain some of the contrasting findings [13,17]. While all studies evaluated performance outcomes, many did not use specific questionnaires to evaluate the incidence and severity of side effects associated with capsaicin and capsiate supplementation. One study incorporated a pilot evaluation of side effects among 4 participants and noted that none of them experienced any “hot” sensations or gastrointestinal distress [14]. Another study used a questionnaire to evaluate the incidence of side effects such as gastrointestinal distress, tachycardia, and headache and reported a very low occurrence of side effects associated with capsaicin ingestion [23]. Still, as this was used only in a single study, future research on the safety of capsaicin and capsiate supplementation is needed. Finally, data from both capsaicin and capsiate supplementation were included in the analysis, given that capsiate is a capsaicin analog, they have a similar chemical structure and mechanism of action [1]. Still, this may be considered a limitation of the review and future studies may consider directly comparing the effects of capsaicin and capsiate on exercise performance.

## 5. Conclusions

This review explored the effects of capsaicin and capsiate on endurance performance using a meta-analysis. Fourteen studies were included in the review, most of which provided capsaicin or capsiate in the dose of 12 mg, 45 min before exercise. There was no significant difference between the placebo and capsaicin/capsiate conditions for aerobic endurance. However, capsaicin/capsiate ingestion was ergogenic for muscular endurance. These ergogenic effects were present even when only isolated capsaicin was considered in the analysis. In addition, an ergogenic effect of capsaicin/capsiate for muscular endurance was found in resistance exercise sets 1, 2, and 3. Capsaicin/capsiate ingestion reduced RPE following muscular endurance but not aerobic endurance tests. In summary, capsaicin/capsiate supplementation acutely enhances muscular endurance coupled with a reduction in RPE, while the effects on aerobic endurance are less clear.

## Figures and Tables

**Table 1:** Summary of the included studies.

**Studies on the Effects of Capsaicin and Capsiate on Aerobic Endurance**			
**Reference**	**Participants**	**Supplementation Protocol**	**Performance Test**
Costa et al. [12]	12 physically active males	12 mg of capsiate 45 min before exercise	400-m and 3000-m running
de Freitas et al. [15]	10 physically active males	12 mg of capsaicin 45 min before exercise	1500-m running
de Freitas et al. [16]	13 physically active males	12 mg of capsaicin 45 min before exercise	Interval running to exhaustion
Giuriato et al. [19]	10 young males	2 × 3.9 mg of capsaicin 50 min before exercise	Cycling to exhaustion at 85% of maximal work rate
Langan et al. [21]	13 recreationally active females and males	1.2 mg of capsaicin 45 min before exercise	Cycling to exhaustion at 90% of VO_2max_
Padilha et al. [22]	15 recreationally trained male runners	12 mg of capsaicin 45 min before exercise	Running to exhaustion at 90% of VO_2peak_
von Ah Morano et al. [25]	21 male amateur athletes	12 mg of capsiate 45 min before exercise	10-km cycling time-trial
**Studies on the Effects of Capsaicin and Capsiate on Muscular Endurance**			
**Reference**	**Participants**	**Supplementation Protocol**	**Performance Test**
Cross et al. [13]	9 recreationally active females and males	1.2 mg of capsaicin 45 min before exercise	120 maximal isokinetic knee extensions
de Freitas et al. [14]	10 resistance-trained males	12 mg of capsaicin 45 min before exercise	4 sets of squats to muscular failure with 70% of 1RM
de Freitas et al. [17]	11 resistance-trained males	12 mg of capsaicin 45 min before exercise and 12 mg of capsaicin immediately before exercise	4 sets of squats to muscular failure with 70% of 1RM performed after a 5-km run
de Moura et al. [18]	20 resistance-trained males	6 or 12 mg of capsiate 45 min before exercise	4 sets of bench press to muscular failure with 70% of 1RM
Gomes et al. [20]	13 resistance-trained males	12 mg of capsiate 45 min before exercise	Five sets of 10-s knee extension MVC
Simoes et al. [23]	11 resistance-trained males	12 mg of capsaicin 45 min before exercise	4 sets of squats to muscular failure with 70% of 1RM
Volino-Souza et al. [24]	8 resistance-trained males	12 mg of capsaicin 45 min before exercise	3 sets of leg extension to muscular failure at 70% of 1RM

1RM: one-repetition maximum; MVC: maximal voluntary contraction; VO_2max_: maximal oxygen consumption; VO_2peak_: peak oxygen uptake.

**Table 2:** Evaluation of risk of bias among the included studies using the risk of bias (RoB) tool 2 for crossover trials.

Reference	Domain 1	Domain S	Domain 2	Domain 3	Domain 4	Domain 5	Overall
Costa et al. [12]	Some concerns	Low risk	Low risk	Low risk	Some concerns	Low risk	Some concerns
Cross et al. [13]	Some concerns	Low risk	Low risk	Low risk	Some concerns	Low risk	Some concerns
de Freitas et al. [14]	Some concerns	Low risk	Low risk	Low risk	Low risk	Low risk	Some concerns
de Freitas et al. [15]	Some concerns	Low risk	Low risk	Low risk	Some concerns	Low risk	Some concerns
de Freitas et al. [16]	Some concerns	Low risk	Low risk	Low risk	Some concerns	Low risk	Some concerns
de Freitas et al. [17]	Some concerns	Low risk	Low risk	Low risk	Some concerns	Low risk	Some concerns
de Moura et al. [18]	Low risk	Low risk	Low risk	High risk	Some concerns	Low risk	High risk
Giuriato et al. [19]	Some concerns	Low risk	Some concerns	Low risk	Some concerns	Low risk	Some concerns
Gomes et al. [20]	Low risk	Low risk	Low risk	Low risk	Some concerns	Low risk	Some concerns
Langan et al. [21]	Some concerns	Low risk	Some concerns	Low risk	Some concerns	Low risk	Some concerns
Padilha et al. [22]	Some concerns	Low risk	Low risk	Low risk	Some concerns	Low risk	Some concerns
Simoes et al. [23]	Some concerns	Low risk	Low risk	Low risk	Some concerns	Low risk	Some concerns
Volino-Souza et al. [24]	Some concerns	Some concerns	Low risk	Low risk	Some concerns	Some concerns	Some concerns
von Ah Morano et al. [25]	Low risk	Some concerns	Low risk	Some concerns	Some concerns	Some concerns	Some concerns

Domain 1—bias arising from the randomization process; Domain S—bias arising from period and carryover effects; Domain 2—bias due to deviations from intended intervention; Domain 3—bias due to missing outcome data; Domain 4—bias in measurement of the outcome; Domain 5—bias in selection of the reported result.

## Data Availability

Data used for the analysis are available from the corresponding authors on request.
